# Enhanced terahertz conductivity in ultra-thin gold film deposited onto (3-mercaptopropyl) trimethoxysilane (MPTMS)-coated Si substrates

**DOI:** 10.1038/s41598-019-51085-0

**Published:** 2019-10-21

**Authors:** Youjin Lee, Dasom Kim, Jeeyoon Jeong, Jugyoung Kim, Volodymyr Shmid, Oleg Korotchenkov, Parinda Vasa, Young-Mi Bahk, Dai-Sik Kim

**Affiliations:** 1 Department of Physics and Astronomy, Seoul National University, Seoul, 08826 Republic of Korea; 2 Department of Physics and Center for Atom Scale Electromagnetism, Ulsan National Institute of Science and Technology, Ulsan, 44919 Korea; 3 Department of Physics, Incheon National University, Incheon, 22012 Republic of Korea; 4 Faculty of Physics, Taras Shevchenko Kyiv National University, Kyiv, 01601 Ukraine; 5 Department of Physics, Indian Institute of Technology Bombay, Mumbai, 400 076 India

**Keywords:** Nanophotonics and plasmonics, Nanophotonics and plasmonics

## Abstract

Various material properties change considerably when material is thinned down to nanometer thicknesses. Accordingly, researchers have been trying to obtain homogeneous thin films with nanometer thickness but depositing homogeneous few nanometers thick gold film is challenging as it tends to form islands rather than homogenous film. Recently, studies have revealed that treating the substrate with an organic buffer, (3-mercaptopropyl) trimethoxysilane (MPTMS) enables deposition of ultra-thin gold film having thickness as low as 5 nm. Different aspects of MPTMS treatment for ultra-thin gold films like its effect on the structure and optical properties at visible wavelengths have been investigated. However, the effect of the MPTMS treatment on electrical conductivity of ultra-thin gold film at terahertz frequency remains unexplored. Here, we measure the complex conductivity of nanometer-thick gold films deposited onto an MPTMS-coated silicon substrate using terahertz time-domain spectroscopy. Following the MPTMS treatment of the substrate, the conductivity of the films was found to increase compared to those deposited onto uncoated substrate for gold films having the thickness less than 11 nm. We observed 5-fold enhancement in the conductivity for a 7 nm-thick gold film. We also demonstrate the fabrication of nanoslot-antenna arrays in 8.2-nm-thick gold films. The nanoslot-antenna with MPTMS coating has resonance at around 0.5 THz with an electric field enhancement of 44, whereas the nanoslot-antenna without MPTMS coating does not show resonant properties. Our results demonstrate that gold films deposited onto MPTMS-coated silicon substrates are promising advanced materials for fabricating ultra-thin terahertz plasmonic devices.

## Introduction

Though a considerable volume of literature has been published about material properties of metallic films^1,2^, properties of ultra-thin metal films having thickness of few nanometers continue to attract attention. The reason for this interest is the significant deviation in physical as well as chemical properties of metal films compared to bulk properties when their thickness is reduced to few nanometers^3–5^. Particularly, the optical properties of thin gold film are important due to their application in fabricating metamaterials and nanoplasmonic devices^6^. Recently, the thickness of devices has become even thinner. In case of ten-nanometer-thick devices, our previous work^7^ has demonstrated that performance, e.g., field enhancement, of nano-plasmonic devices, is increased by 6-fold, compared to a hundred-nanometer-thick devices. Hence, fabrication and comprehensive investigation of optical properties of ultra-thin gold film are promising as well as essential.

Interestingly, ultra-thin gold films (~several nanometers), exhibit a metal-to-insulator percolation transition^8,9^. Although electron-beam (e-beam) and thermal evaporators enable deposition of gold layer having thickness down to few nanometers, the layer is highly inhomogeneous. The fabrication process generally gives rise to nanometer-sized isolated gold islands before a homogeneous layer is deposited^10^. This limits the lowest thickness of the continuous film that can be deposited, which in turn limits the size-reduction of nano-devices. Several methods to make continuous films have been demonstrated and developed^11,12^. One of the possibility is a deposition of a primer layer^11^, such as Ti, Ge, and Cr, on Si substrate followed by the deposition of gold but the primer atoms diffuse into gold contaminating the film^13,14^. The other possibility^12^ is a template-stripped method but it gives rise to imperfect stripping^15^. Several studies have reported introducing an organic buffer called (3-mercaptopropyl) trimethoxysilane (MPTMS) layer between the gold film and the substrate^16^. The organic buffer allows deposition of continuous and homogeneous gold films below the metal-to-insulator percolation transition^10^. Subsequently, the enhanced optical properties of ultra-thin gold film are investigated in visible frequencies^10^. Nevertheless, they are not yet investigated in terahertz frequency range, which is an important spectral range for fabricating plasmonic and nano-photonic devices. As the real and imaginary parts of electrical conductivity can be effectively extracted by performing time-domain spectroscopy, studying optical properties also helps in understanding carrier dynamics in these thin films. In this spectral range, the performance of plasmonic devices, e.g. field enhancement, is larger than that of the visible regime^17^ because the permittivity of gold is hundred thousand times larger than that at visible frequencies.

In this paper, we investigate the enhanced terahertz conductivity of ultra-thin gold films deposited onto MPTMS-coated silicon substrates. To compare complex conductivity of gold films deposited onto silicon substrate with and without MPTMS treatment, we perform terahertz time-domain spectroscopy from 0.4 to 2.0 THz. Subsequent DC conductivity measurement is also performed to confirm our results. Our results reveal that 5.8-nm-thick gold film having metallic character can be deposited using evaporation onto MPTMS-coated substrates, whereas those deposited onto bare substrates exhibit dielectric character. Thus, the thickness threshold for metal to insulator transition is significantly reduced for gold films deposited onto the MPTMS-coated substrates. We also, observe a 5-fold enhancement in conductivity for a 7 nm-thick film. Similar increase is also observed in terahertz field enhancement of metal nanoslot antennas following MPTMS treatment. Our results demonstrate enhanced metallic performance of ultra-thin gold films, which is essential for fabricating nano-devices. Thus, pre-treatment of substrates for depositing ultra-thin gold films will open up several possibilities of terahertz metamaterials and plasmonic devices comprising ultra-thin gold films.

## Results and Discussion

**Figure 1:**
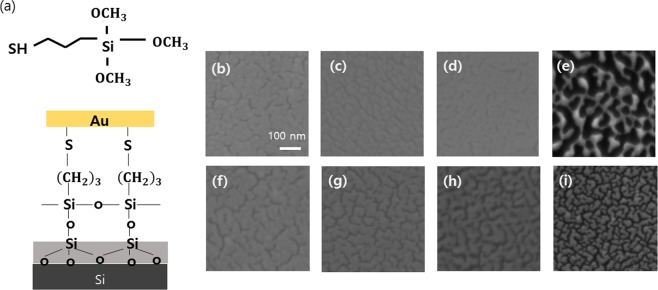
A schematic diagram showing MPTMS structure (**a**, top) and gold film on silicon oxide layer with MPTMS adhesive layer (**a**, bottom). SEM images of thin gold films deposited onto MPTMS-coated (top row) and uncoated (bottom row) Si substrates. The gold films have a of 10.5 nm (**b**,**f**), 8.2 nm (**c**,**g**), 7.6 nm (**d**,**h**), and 5.8 nm (**e**,**i**).

We deposited ultra-thin gold films on silicon substrates using e-beam evaporation technique. Before the deposition, the substrates were prepared as follows: The substrates were cleaned in an ultrasonic bath with acetone and IPA solution for 5 minutes and dried with nitrogen gas. Subsequently, oxygen plasma treatment was carried out with RF power of 300 W, base pressure of 80 mTorr, and O_2_ flow rate of 200 sccm. After that, the substrates were exposed to vapor-phase MPTMS at room temperature over 48 hours under pressure of 310 Torr. This duration was sufficient to complete the silanization process of silicon using MPTMS under low pressure environment^18^. The MPTMS treatment is schematically shown in Fig. 1(a). Two functional groups in MPTMS, trimethoxysilane and thiol group, work as a bridge between gold atoms and the substrate. The trimethoxysilane function group reacts with silica surfaces forming a monolayer. The thiol group (-SH) at the other end of MPTMS molecules is toward the air, which release hydrogen gas after the reaction with gold. The gold was deposited on MPTMS-coated and uncoated substrate simultaneously by e-beam evaporator. The evaporation rate was 1 Å/s and a base pressure was below ${10}^{-6}$ Torr. The film thickness is measured by a quartz crystal microbalance during the deposition, which was previously calibrated by atomic force microscope (AFM).

The deposition of the ultra-thin gold film with MPTMS coating and e-beam evaporator has some advantages over other standard deposition techniques. One is the ease of fabrication. All process is the same with pristine E-beam evaporation method except for MPTMS coating. Moreover, the e-beam evaporation of gold film is prevalent method compared with the metal atomic layer deposition (ALD) and metal chemical vapor deposition (CVD). Another is flexibility in lithographic processes. For ALD or CVD, the gold film deposition should precede the lithographic process, due to the risk of contamination from resist. Therefore, a gold film can be patterned via etching method. On the other hand, e-beam evaporation with MPTMS coating is compatible with either lift-off or etching method. Therefore, this e-beam evaporation with MPTMS coating is more suitable to fabricate ultrathin terahertz metamaterial and plasmonic devices. The MPTMS coating is efficient with some physical vapor depositions (PVD). Sputtering is compatible with e-beam evaporation. Not all PVD, however, is efficient method due to UV activation of MPTMS^10^. For e-beam evaporation and sputtering, the MPTMS coated substrate is exposed to UV radiation during evaporation. This high-energy radiation weakens the bond between sulfur and hydrogen at the end of the MPTMS molecule. This photochemical reaction increases the reaction between sulfur and gold. In the case of thermal evaporation, the substrate cannot be exposed to UV. Therefore, an additional exposure step needs before deposition.

Figure 1(b–i) correspond to scanning electron microscopy images showing the surface morphology of the gold films. The morphology is strongly dependent with film thickness^19^. In the early stage of the gold deposition, the gold particles form disconnected island due to their surface diffusion. With continued deposition, the islands increase in size and then coalesce to form a network. At the thickness of 5.8 nm, the MPTMS-coated film (Fig. 1(e)) has connected gold clusters forming a network, on the other hand, the uncoated film (Fig. 1(i)) has isolated clusters. In the thicker thickness, the MPTMS-coated films (Fig. 1(b–d)) are more continuous with fewer voids than the uncoated films (Fig. 1(f–h)). This improvement of MPTMS-coated films is because the reaction with MPTMS reduces surface diffusion of gold, producing nucleation sites and leading to the laterally continuous film^10^. In a thin metal film, the voids cause the electrons to scatter back at the boundary of the gold clusters. The backscattering degrades the conductivity of a gold film. This implies that MPTMS-coated films have enhanced conductivities than uncoated films.

**Figure 2:**
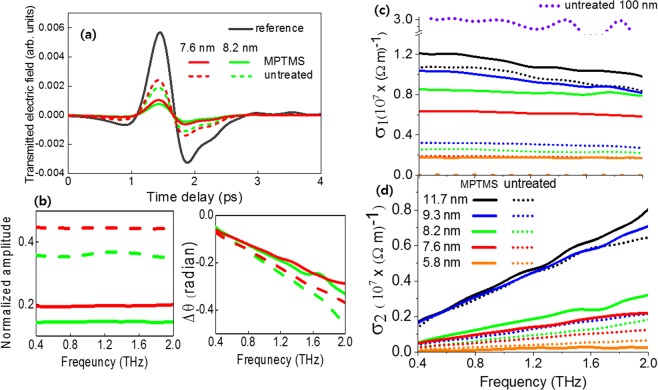
(**a**) Time traces of transmitted terahertz electric field through 7.6 nm (red) and 8.2 nm (green) thick gold films on Si substrates and through bare Si substrate as a reference. Solid line and dotted line represent MPTMS-coated samples and uncoated samples respectively. (**b**) The corresponding Fourier-transformed amplitudes (left) and phases (right). Real (**c**) and imaginary (**d**) part of conductivity for ultra-thin gold films having different thicknesses. The data clearly reveal that the MPTMS-coated films (solid line) have enhanced conductivity than uncoated films (dotted line) at same thickness.

To examine electrical property of ultra-thin gold films in terahertz frequency, we extracted complex electrical conductivity via terahertz time-domain spectroscopy. Both amplitude and phase of electric field can be obtained by direct measurement of time traces. Brief explanation of the set-up is as follow. A single cycle of terahertz pulse is generated from a biased LT-GaAs emitter illuminated by a femtosecond Ti:sapphire laser with center wavelength of 800 nm, repetition rate of 80 MHz and pulse width of 130 fs. The terahertz pulse is focused onto the sample by off-axis parabolic mirror. A transmitted electric field is detected by electro-optic sampling technique using a ZnTe crystal. Figure 2(a) shows time traces of transmitted electric fields through the ultra-thin gold films on substrate. It clearly shows that transmission is suppressed in case of MPTMS-coated samples. We extracted complex electric fields in frequency-domain by Fourier transformation shown in Fig. 2(b), to calculate the refractive indices in frequency-domain. The relation between the electric fields and the refractive indices is given as^20^

$$\frac{{\tilde{E}}_{film+substrate}(\omega )}{{\tilde{E}}_{substrate}(\omega )}=\frac{{t}_{12}\,{t}_{23}\,exp[i{k}_{0}{n}_{2}h]}{(1+{r}_{12}\,{r}_{23}\,exp[2i{k}_{0}{n}_{2}h]){t}_{13}\,exp[i{k}_{0}{n}_{1}h]};$$

where *t*_ij_ and *r*_ij_ are complex Fresnel transmission and reflection coefficients, respectively, between medium *i* and *j*. Here, *n*_i_ is refractive index of medium (*i* = 1, 2, 3 for air, ultra-thin gold film with thickness *h*, silicon substrate, respectively), with *n*_1_ = 1 and *n*_3_ = 3.4. We used a bare silicon substrate as a reference for MPTMS-coated and uncoated films. The values of the transmitted electric field of MPTMS-coated silicon and bare silicon are same, (as shown in Fig. S1 of Supplementary Information), which means that all transmission values normalized by bare silicon substrate provide the same results with that normalized by MPTMS-coated silicon. Using the extracted indices, the complex conductivity of the gold film is obtained by the relation^21^, ${[{n}_{2}(\omega )]}^{2}=1+\frac{i\sigma (\omega )}{{\varepsilon }_{0}\omega }$. The complex conductivity can be also extracted from Tinkham formula^22^:

$$\frac{{\tilde{E}}_{film+substrate}(\omega )}{{\tilde{E}}_{substrate}(\omega )}=\frac{1+{n}_{3}}{1+{n}_{3}+{Z}_{0}\tilde{{\rm{\sigma }}}(\omega )h};$$

where Z_0_ is the impedance of free space. Tinkham formula gives the same complex conductivity values with the above calculation (see Supplementary Fig. 2). Figure 2(c,d) show real and imaginary parts of conductivity for films with varying thickness. Solid lines represent conductivity of MPTMS-coated films, whereas dotted lines represent those of uncoated films. The real part of conductivity is nearly frequency-independent, and the imaginary ones linearly increase with frequency. As expected, and also seen in Fig. 2(c,d), the conductivity decreases with the reduction in thickness. In thinner film, electron scatterings at surfaces and grain boundaries would be much more efficient, resulting in lowering conductivity. The uncoated film is insulating in nature $({\sigma }_{1}\simeq 0)$ at the thickness of 5.8 nm, while MPTMS-coated film remains conducting ($({\sigma }_{1}\simeq 1.7\times {10}^{6}\,{(\varOmega m)}^{-1}$). Our observations show that the threshold for metal to insulator transition is lowered than at least the thickness of 5.8 nm when the substrates are coated with MPTMS.

**Figure 3:**
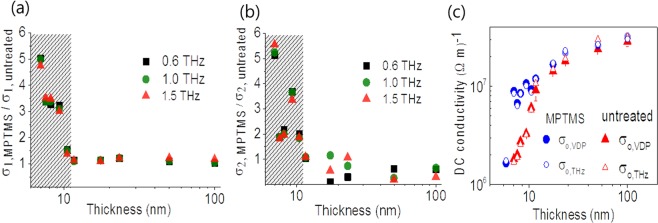
The ratio of real (**a**) and imaginary (**b**) parts of conductivity of the MPTMS-coated samples to the uncoated samples at different frequencies; 0.6 THz (black), 1 THz (green), 1.5 THz (red). The thicknesses of films are 7 nm, 7.6 nm, 8.2 nm, 9.3 nm, 10.5 nm, 11.7 nm, 17. 5 nm, 23.3 nm, 50 nm, and 100 nm. The ratio of 5.8 nm is not plotted in (**a**,**b**), because the ratio of real conductivity goes infinite (${\sigma }_{1}\simeq 0$ for the uncoated film). As the film becomes thinner than 11 nm, the ratio increases rapidly (gray dashed region). (**c**) DC conductivity of MPTMS-coated (blue) and uncoated gold films (red) from Van der Pauw measurements and THz transmission experiments. The film plotted in Fig. 3. (**a**,**b**) are described in addition with 5.8 nm MPTMS coated-film. DC conductivity values extracted from two different measurements agree well within the error range.

The MPTMS-coated films have larger conductivity than those of the uncoated films at the same thickness. The ratios of the real and imaginary part of conductivities of those films are presented in Fig. 3(a,b). The conductivities of MPTMS-coated films are much larger than those of the uncoated ones at the thickness lower than 11 nm. The conductivity of MPMTS-coated one is five times larger than that of uncoated one at the thickness of 7 nm. For lower thickness values, the films deposited on uncoated substrates are insulating. When gold islands partly coalesce to form semi-continuous film, conduction generally takes place via two processes: (i) metallic conduction through complete links and (ii) thermally activated tunneling through isolated metal islands. As more links between the islands form, the conductivity increases. In this case, our results suggest the higher conductivity due to the enhanced lateral continuity in MPTMS-coated film. As shown in Fig. 1(b–e), in MPTMS-coated sample, gold islands have connected network even at the thickness of 5.8 nm. For larger thickness, they are fully connected with each other forming continuous film. In contrast, for uncoated sample shown in Fig. 1(f–i), gold islands are disconnected for the thickness of 5.8 nm. Also, the islands remain only partially connected with each other even above the thickness of 5.8 nm.

To confirm the conductivity value obtained from terahertz time-domain spectroscopy, we compare the DC conductivities (*σ*_0_) derived from terahertz conductivity ($\tilde{\sigma }$) with and the ones obtained by Van der Pauw method. The relation we used is

$$\tilde{\sigma }={\sigma }_{1}+i{\sigma }_{2}=\frac{{\sigma }_{0}}{1-i\omega \tau },$$

where *τ* is the electron scattering time for gold. DC conductivity was measured by a commercial Hall measurement system, HL5500PC (BIO-RAD Co.). We used the Van der Pauw configuration for resistivity measurement. And then we converted resistivity to conductivity. Before resistivity measurement, we made the contact composed with a silver paste at the corner of the rectangle-shaped gold film. Figure 3(c) shows the DC conductivity as a function of the film thickness. The two conductivity values obtained from different measurements are consistent within the error range. The DC conductivity of the thickest film, 100 nm, is $3.19\times {10}^{7}{(\varOmega m)}^{-1}$. This value is lower than $4.52\times {10}^{7}\,{(\varOmega m)}^{-1}$, the literature value for single crystalline bulk gold^23^. Such low conductivity values are common for evaporated metal films and have been attributed to a polycrystalline film growth^24^.

Here, we also demonstrate that ultra-thin gold film deposited onto MPTMS-coated substrates can improve the performance of plasmonic devices. Among the plasmonic devices, nanoslot antenna is becoming increasingly popular due to its large near-field enhancement factors^25^. When light is incident to a nanoslot antenna patterned on the thin metal film, charges are accumulated at the edges of the metal gap via light-induced currents in the metal film. This charge accumulation creates a strong electric field inside and near the gap. It had been reported that the near-field enhancement of 1 nm-wide slot antenna reaches up to 25,000 in terahertz frequency regime^26^.

**Figure 4:**
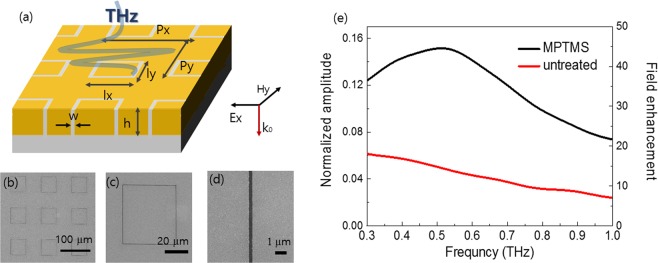
(**a**) Terahertz pulse is incident onto the ring-shaped nano-slot antenna structure. (b-d) Top-view of SEM images for ring-shaped nano-slots. (gold thickness *h* = 8.2 *nm*; gap width *w* = 340 *nm*; *l*_*x*_ = *l*_*y*_ = 50 *μm*; *p*_*x*_ = *p*_*y*_ = 100 *μm*). (e) Normalized transmission amplitude and field enhancement spectra of the nanoslot array. The direct transmission of film is subtracted from slot array data. Field enhancement factor reaches 44 at 0.5 THz in MPTMS-coated sample, whereas uncoated sample has no distinct peak.

We fabricated ultra-thin ring-shaped nanoslot antenna for terahertz frequencies. The patterns were fabricated by e-beam lithography onto the MPTMS-coated substrate and the bare substrate. Subsequently gold was deposited using e-beam evaporation and the nanoslot antenna was fabricated after completing the lift-off process. Figure 4(a–d) show the experimental scheme and the SEM images of the samples having parameters of gold thickness *h* = 8.2 nm, gap width *w* = 340 nm, *l*_x_ = *l*_y_ = 50 μm, and *p*_x_ = *p*_y_ = 100 μm.

The strong electric field inside the metal gap becomes a dipole source, resulting in an enhanced far field transmission through the metal nanogap^27^. According to Kirchhoff integral formalism, the near field enhancement, ${E}_{near}^{slot}/{E}_{near}^{aperture}$ can be estimated by following equations,

$$|\frac{{E}_{far}^{slot}}{{E}_{far}^{aperture}}|=\frac{\frac{{e}^{ikR}}{i\lambda R}\int {E}_{near}^{slot}d{A}_{slot}}{\frac{{e}^{ikR}}{i\lambda R}\int {E}_{near}^{aperture}d{A}_{aperture}}=\frac{{E}_{near}^{slot}{A}_{slot}}{{E}_{near}^{aperture}{A}_{aperture}}$$

where *A*_slot_ and *A*_aperture_ are areas of metal gap and aperture, respectively. Finally, with the measured far field transmission |*E*^slot^_far_/*E*^aperture^_far_| and the area-coverage ratio of the nanogap relative to the total illuminated area *A*_slot_/*A*_aperture_, we can estimate the field enhancement factor. In order to take into account the propagating near field only from the metal gap region, we subtracted the transmitted amplitude through the bare gold film from that through metal nanogap samples. The transmitted terahertz signals through the metal nanogaps are normalized by the reference signal which is the transmitted amplitude through bare silicon onto 2 mm by 2 mm aluminum aperture.

The transmission spectrum of MPTMS-coated metal nanogap represents resonant excitation of the fundamental waveguide mode with an electric field enhancement of 44 at the resonance, while that of uncoated sample does not show resonant properties. This can be explained by degradation of conductivity in uncoated metal films, resulting in not enough current flow in a metal film to make charge accumulation. In conclusion, we demonstrate that MPTMS-treatment enables the sub-10nm thick plasmonic devise to act as terahertz antenna.

## Conclusion

We have fabricated ultra-thin gold films by evaporation onto MPTMS-coated silicon substrates. We have characterized these films structurally as well as measured the complex terahertz conductivity using terahertz time-domain spectroscopy and compared it to gold films deposited onto uncoated substrates. Our results show that MPTMS treatment significantly helps in fabricating continuous and homogeneous gold films. The treatment also lowers the thickness threshold for metal-to-insulator percolation transition and results in the 5-fold enhancement of THz conductivity. We also fabricated sub-10 nm thick THz active nano-slot antennas exhibiting 44-fold increase in field enhancement. We anticipate that our observations will contribute towards establishing ultra-thin gold films as promising material for fabricating terahertz metamaterials and plasmonic devices.

## Supplementary information

SUPPLEMENTARY INFORMATION
