# Supplementary material for: Enhanced terahertz conductivity in ultra-thin gold film deposited onto (3-mercaptopropyl) trimethoxysilane (MPTMS)-coated Si substrates
Source: Sci Rep. 2019 Oct 21;9:15025. doi: 10.1038/s41598-019-51085-0 (PMC6803624; doi:10.1038/s41598-019-51085-0)
Supplement: Supplementary file 1 — SUPPLEMENTARY INFORMATION [file 41598_2019_51085_MOESM1_ESM.pdf]

SUPPLEMENTARY INFORMATION

Enhanced terahertz conductivity in ultra-thin gold film deposited onto (3-mercaptopropyl) trimethoxysilane (MPTMS)-coated Si substrates

Youjin Lee[§1], Dasom Kim[§2], Jeeyoon Jeong[1], Jugyoung Kim[3], Volodymyr Shmid[4], Oleg Korotchenkov[4], Parinda Vasa[5], Young-Mi Bahk*[3], Dai-Sik Kim*[1,2]

[1]Department of Physics and Astronomy, Seoul National University, Seoul 08826, Korea

[2]Department of Physics and Center for Atom Scale Electromagnetism, Ulsan National Institute of Science and Technology, Ulsan 44919, Korea

[3]Department of Physics, Incheon National University, Incheon 22012, Korea

[4]Faculty of Physics, Taras Shevchenko Kyiv National University, Kyiv 01601, Ukraine

[5]Department of Physics, Indian Institute of Technology Bombay, Mumbai 400 076, India

[§]Y.L. and D.K. contributed equally to this work

*E-mail: ymb@inu.ac.kr

*E-mail: daisikkim@unist.ac.kr

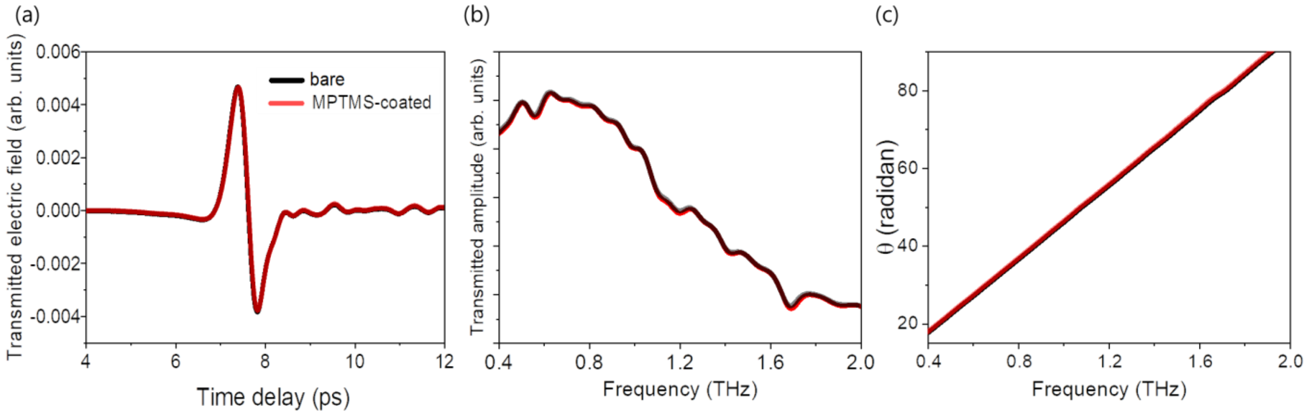

Figure S1. MPTMS-coated silicon substrate before gold deposition. (a) Time traces of transmitted terahertz electric field through the bare (black) and MPTMS-coated (red) silicon substrates. The corresponding Fourier transformed amplitudes (b) and phases (c). Within the measurement error, the MPTMS-coated silicon substrate has the same values with the bare silicon substrate.

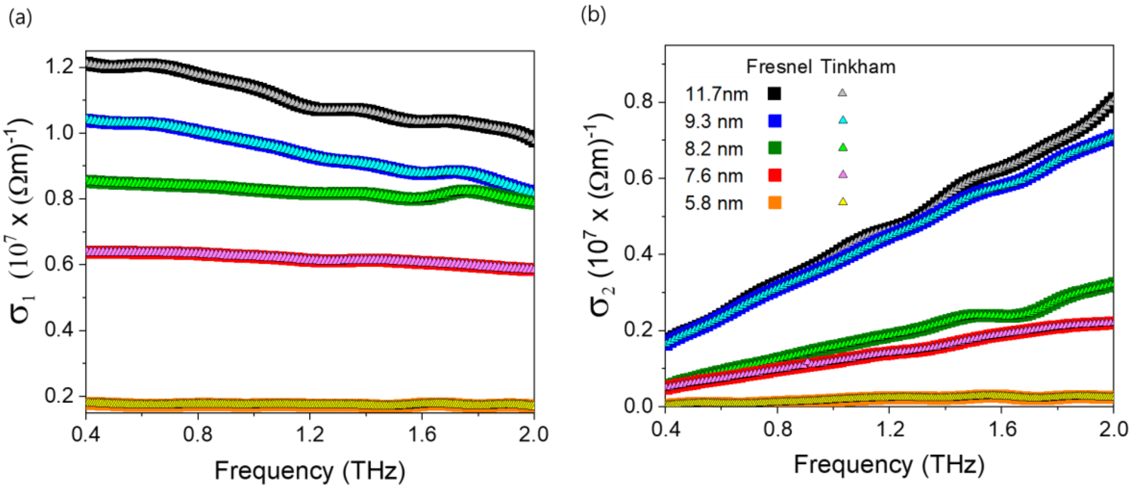

Figure S2. Comparison of conductivities from Fresnel coefficient formula and Tinkham formula. Real (a) and imaginary (b) part of conductivity for different thicknesses. The data clearly reveal that the conductivity from Tinhkam (triangle) is equal to the conductivity (rectangle) from the Fresnel coefficient formula at the same thickness and frequency.
